# Heart Failure With Reduced Ejection Fraction Caused by Osimertinib in a Patient With Lung Cancer: A Case Report and Literature Review

**DOI:** 10.7759/cureus.27694

**Published:** 2022-08-05

**Authors:** Shinichi Okuzumi, Masahiro Matsuda, Genta Nagao, Tomoo Kakimoto, Naoto Minematsu

**Affiliations:** 1 Department of Medicine, Hino Municipal Hospital, Hino-shi, JPN

**Keywords:** echocardiogram, tyrosine kinase inhibitor, epidermal growth factor receptor, adenocarcinoma, lung cancer, cardiotoxicity, heart failure, osimertinib

## Abstract

Osimertinib is widely used for the treatment of advanced lung cancers harboring *epidermal growth factor receptor* (*EGFR*) mutations. Because of its inhibitory activity on the human epidermal growth factor receptor 2 pathway, osimertinib-induced cardiotoxicity is concerning. Large-scale international clinical studies revealed a subclinical decline in the left ventricular ejection fraction (LVEF) with osimertinib, which allowed a continuation of the drug. Only a few studies have reported symptomatic heart failure with reduced ejection fraction (HFrEF) with osimertinib, and its clinical impact in real-world settings remains unclear. A 91-year-old man was diagnosed with lung adenocarcinoma harboring an *EGFR L858R *mutation and was started on osimertinib. The treatment conferred substantial tumor regression; however, the patient presented with symptomatic HFrEF six weeks after osimertinib initiation. Transthoracic echocardiography demonstrated diffuse hypokinesis of the left ventricular walls with a significantly reduced ejection fraction from the baseline. Initial evaluation showed no causative cause of heart failure, and we suspected osimertinib-associated cardiomyopathy. Discontinuation of the drug along with the cardioprotective approach improved cardiac symptoms and restored the LVEF to baseline within a week. Here, we comprehensively review the literature and discuss the clinical features of HFrEF following osimertinib administration. Physicians should be aware of rare complications associated with osimertinib therapy.

## Introduction

Osimertinib, a third-generation epidermal growth factor receptor (EGFR)-tyrosine kinase inhibitor (TKI), is widely used to treat advanced lung cancer harboring *EGFR*-sensitive mutations [1]. It also inhibits the human epidermal growth factor receptor 2 (HER2) pathway and potentially causes cardiomyopathies [2]. In large-scale international clinical studies with osimertinib, surveillance of transthoracic echocardiogram (TTE) estimated the incidence of reduced left ventricular ejection fraction (LVEF) to be 4.3% [3]. These patients were mostly asymptomatic and allowed to continue osimertinib treatment. On the contrary, a few studies have reported symptomatic heart failure with reduced ejection fraction (HFrEF) associated with osimertinib. The clinical impact of osimertinib on cardiomyopathy in real-world settings remains unclear. Here, we report a case of symptomatic HFrEF following osimertinib treatment initiation in a patient with advanced lung adenocarcinoma along with a literature review. This study contributes to the literature regarding the accumulation of cardiomyopathies caused by osimertinib.

The abstract of this case was presented at the regional meeting of the Japanese Respiratory Society in 2021.

## Case presentation

**Figure 1:**
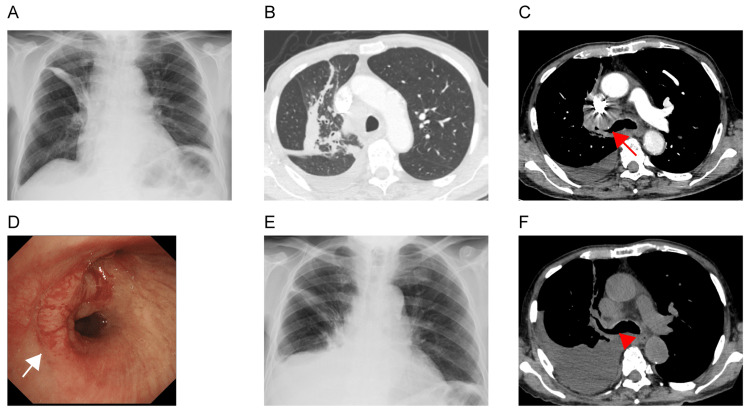
Radiological and bronchoscopic findings on the first and second admission. Chest radiograph showing band-like opacities in the right upper lung field (A). Chest computed tomography (CT) revealed a hilar tumor that had invaded and mostly obstructed the right upper lobe bronchus (arrow). Interlobar pleural effusion is present on the right side (B and C). Bronchoscopy revealed tumor invasion in the right main (arrow) and upper lobe bronchi (D). The right-sided pleural effusion increased six weeks after initiating osimertinib treatment (E). Chest CT demonstrating a decreased hilar tumor and a secured right upper lobe bronchus (arrowhead) (F).

A 91-year-old man (non-smoker) complained of exertional dyspnea at our hospital. The patient had hypertension and chronic kidney disease and was prescribed 30 mg azosemide and 2 mg tolvaptan for sustained lower extremity edema. Four months prior, the patient had normal left ventricular wall motion with an intact LVEF (66%, modified Simpson’s method) and had no comorbidities relevant to extremity edema. Therefore, the physician diagnosed the patient’s edema as attributable to chronic kidney disease, hypoproteinemia (serum albumin: 3.0 g/dL), and low physical activity in the elderly patient. Chest radiography revealed band-like opacity in the right upper lung field (Figure 1, Panel A). On chest computed tomography (CT), a hilar tumor directly invaded and narrowed the bronchus of the right upper lobe. In addition, CT revealed mediastinal lymph node enlargement, right-sided pleural effusion (Figure 1, Panel B and C), and liver metastasis (not shown). Electrocardiography (ECG) showed normal sinus rhythm without ST-T changes or QT interval prolongation. TTE showed normal wall motion of the left ventricle with an LVEF of 64% and normal chamber calibers that were unchanged from the previous study. Laboratory examination showed a slight increase in brain natriuretic peptide (BNP) (106.1 pg/mL) and serum creatinine (2.07 mg/dL) levels, which were similar to those in the previous examinations. The serum carcinoembryonic enzyme (CEA) level was extremely high (1,086.8 ng/mL). The patient underwent bronchoscopy, and a pathological diagnosis of adenocarcinoma was made based on the biopsy specimens from the right main bronchus (Figure 1, Panel D). Thoracentesis of the right pleural effusion revealed an adenocarcinoma. The cancer cells had an *EGFR* exon *21 L858R* point mutation. We finally diagnosed the patient with lung adenocarcinoma with cT2aN2M1b PLE, HEP, and clinical stage IVA. The patient was started on osimertinib (80 mg/day) and discharged from the hospital.

**Figure 2:**
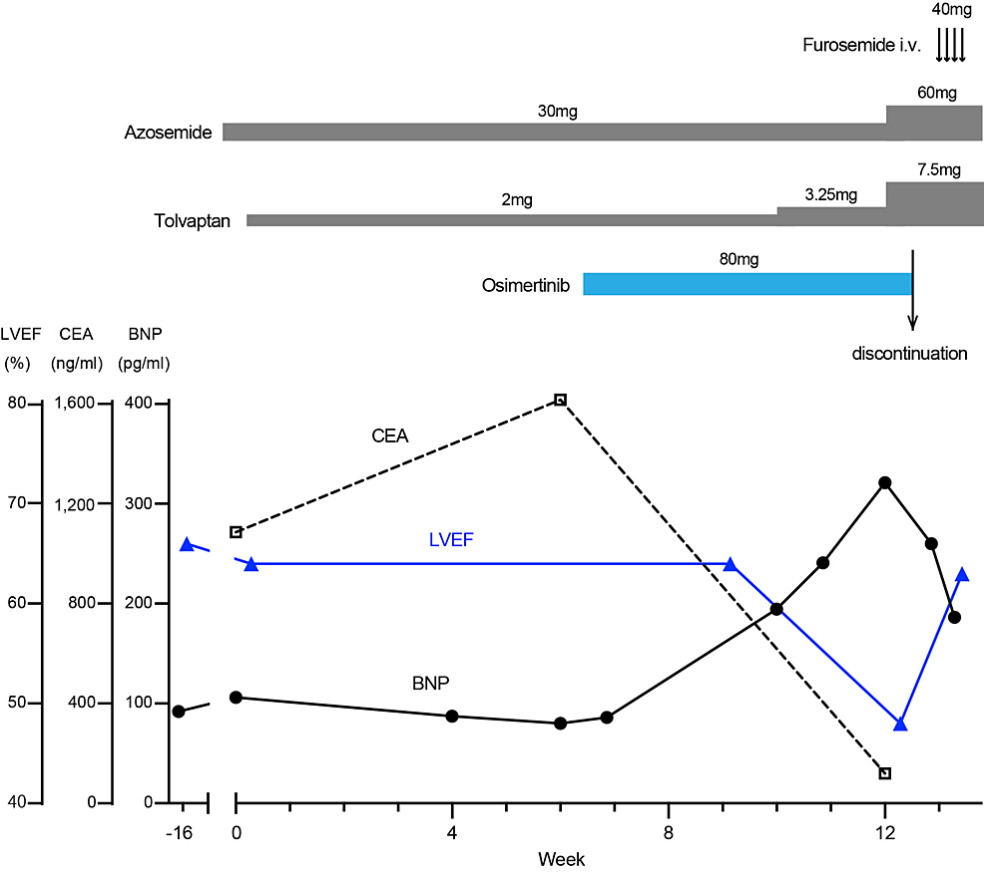
Clinical course. BNP: brain natriuretic peptide; CEA: carcinoembryonic enzyme; LVEF: left ventricular ejection fraction

The BNP level was slightly elevated at three weeks following osimertinib initiation (194.4 pg/mL) without any subjective signs. The patient reported good adherence to medication and a salt-restricted diet. We increased the tolvaptan dose from 2 to 3.25 mg daily and recommended a follow-up visit. However, the patient required a second admission due to progressive exertional dyspnea, increased right-sided pleural effusion, and systemic edema for another three weeks. Physical examination revealed a pulse rate of 69 beats/minute, blood pressure of 159/87 mmHg, and SpO_2_ of 95% on ambient air. The BNP level was elevated to 321.1 pg/mL (Figure 2), and cardiac enzymes were within the normal range. The other laboratory findings were unremarkable. An ECG showed normal sinus rhythm without ST-T changes, arrhythmia, or QT interval prolongation. TTE showed diffuse hypokinesis of the left ventricular wall, with a decreased LVEF to 48% (16% points down). Coronary angiograms and complete evaluation of viral titers were suspended because ischemic heart disease or viral myocarditis was unlikely based on the initial evaluation. The patient was diagnosed with HFrEF with an undefined etiology, independent of progressive hypertension, ischemic heart disease, valvular disease, or arrhythmia. A chest CT showed a decreased hilar tumor and mediastinal lymph nodes, resulting in recanalization of the right upper lobe bronchus (Figure 1, Panel E and F). Thoracentesis of the right-sided pleural effusion revealed slightly exudative effusion (pleural effusion/serum ratio of total protein: 0.53) with negative cytological results. His serum CEA level dramatically decreased to 119.8 ng/mL (Figure 2). These findings showed that osimertinib conferred a substantial tumor regression, but suggestively caused grade 3 cardiomyopathy (Common Terminology Criteria for Adverse Events v5.0). The Naranjo scale score, used for assessment of causality for adverse drug reactions, was three (possible) [4]. Osimertinib was withheld and intravenous furosemide (40mg daily) was administered for four days. In addition, the dosage of azosemide and tolvaptan was increased to 60 and 7.5 mg, respectively. One week after osimertinib discontinuation, the serum BNP level decreased and cardiac symptoms were relieved. In addition, TTE revealed normalization of left ventricular wall motion with full restoration of LVEF up to 63% (Figure 2). The patient refused to resume osimertinib or use other EGFR-TKIs and opted for standard supportive care. The same dose of diuretics was continued and the patient was discharged from the hospital.

## Discussion

The present study reports a patient with symptomatic HFrEF following osimertinib administration for advanced lung cancer. The cardiomyopathy was possibly associated with osimertinib and rapidly improved after its discontinuation, albeit with the escalation of diuretics.

Cardiotoxicity related to anticancer drugs can be classified into two types [5]. Type I toxicities are irreversible myocardial damage caused by anthracyclines in a cumulative dose-dependent manner. On the contrary, type II toxicity indicates reversible myocardial dysfunction via inhibition of the HER2 signaling pathway. Owing to the potent activity of osimertinib in inhibiting the HER2 signaling pathway [2], type II cardiotoxicity has been a target of interest in clinical studies. In the pooled analysis of two large-scale international clinical studies, FLAURA [1] and AURA3 [6], surveillance TTE estimated the incidence of osimertinib-associated LVEF decline as 4.3% (22/512) [3]. It should be noted that 20 of 22 (90.9%) patients were asymptomatic, and 19 (86.4%) patients continued osimertinib without progressing LVEF decline. These findings suggest that osimertinib-associated cardiomyopathies are mostly subclinical and that symptomatic HFrEF is an exception.

To discuss the clinical impact of osimertinib-associated cardiomyopathies in real-world settings, we comprehensively reviewed the English-language literature and found 19 cases of heart failure or relevant cardiac events following osimertinib administration. Among the cases included, 12 cases fulfilled the criteria of LVEF decline on TTE, defined as a decline of more than 10% from a baseline and an absolute value of less than 50% [7]. These criteria were used in international clinical studies [3]. Four cases were excluded from further discussion because of asymptomatic LVEF decline (n = 3) or the coincidence of acute coronary diseases requiring catheter intervention (n = 1). To the best of our search, we found eight cases of symptomatic HFrEF associated with osimertinib (Table 1) [8-12]. One patient had a coincidental pulmonary artery embolism, and three had QT interval prolongation. The predominance of females and non-smoking status (not shown) was reflected in the higher prevalence of *EGFR *mutations in these populations. In all cases, osimertinib was used as first- or second-line therapy for advanced lung adenocarcinoma harboring various *EGFR *mutations. All patients were >70 years old with a median age of 72 years (range = 70-91 years), which was higher than that in the FLAURA [1] and AURA3 [6] studies (64 and 62 years old, respectively). All seven patients (data not available for two cases) had predisposing cardiomyopathy risks, including hypertension, dyslipidemia, ischemic heart disease, and cardiac valve disease. The median time to event was shorter in symptomatic patients with HFrEF (median = 8; range = 2-56 weeks) than in asymptomatic patients with LVEF decline in the AURA3 trial [3] (median = 22 weeks; range = not available). Based on these findings, physicians should monitor cardiac function in elderly patients with cardiac risk factors, particularly in the early phase of osimertinib initiation.

**Table 1:** Cases with symptomatic cardiac failure following osimertinib administration. ACEi: angiotensin-converting enzyme inhibitor; ARB: angiotensin II receptor blocker; Af: atrial fibrillation; CAD: coronary artery disease; CI: cerebral infarction; CKD: chronic kidney disease; HTN: hypertension; HUA: hyperuricemia; LVEF: left ventricular ejection fraction; MR: mitral valve regurgitation; N/A: not available; PCI: percutaneous coronary intervention; PMH: personal medical history; TAA: thoracic aortic aneurysm; TR: tricuspid valve regurgitation. * Time to onset after increasing the dose of osimertinib from 40 mg/day to 80 mg/day.

Age, sex	Race	Cardiac risk	Mutation status	Treatment lines	Time to the event (weeks)	Cardiac event	Treatment for a cardiac event	LVEF (%)				Outcome	Reference
								baseline	at event	After discontinuation (time)	retry		
70, M	Italy	HTN	19 Del T790M	Second	8	Heart failure	Diuretics, ACEi, β-blocker	60	45	48 (3 weeks)	-	Died	[8]
71, F	Italy	N/A	19 Del T790M	Second	44	Heart failure	Diuretics, ACEi, β-blocker	58	45	54 (N/A)	Stable	Alive	[8]
84, F	USA	CAD, CI	L858R	First	4*	Heart failure	Diuretics, ARB, β-blocker	63	20	41 (4 weeks)	-	Alive	[9]
71, M	USA	AF, HTN, DL	G719C S768I	First	2	Heart failure	Diuretics, ACEi, β-blocker	52	39	N/A	Stable	Alive	[9]
72, F	USA	N/A	19 Del	First	4	Heart failure	ACEi, β-blocker	67	38	57 (8 weeks)	-	Alive	[9]
78, F	Japan	HTN, TAA	L858R	Second	12	Heart failure, QT prolongation, MR progression	Diuretics, ARB, β-blocker	61	28	26 (3 months) 48 (9 months)	-	Alive	[10]
84, F	Japan	HTN	19 Del	First	8	Heart failure, QT prolongation	Electrical cardioversion, diuretics, ACEi, β-blocker	65	35	45% (2 weeks) 62% (4 months)	-	Alive	[11]
70, F	USA	Valvular disease	Ex21	First	24	Heart failure, pulmonary embolism, QT prolongation, MR and TR progression	Correction of hypokalemia	55	40	N/A	-	Alive	[12]
91, M	Japan	HTN, CKD	L858R	First	6	Heart failure	Diuretics	64	48	64 (1 week)	-	Alive	Present

As type II cardiomyopathy can be reversible [5], osimertinib-induced cardiomyopathy is expected to improve by drug discontinuation. However, the sole effect of drug discontinuation on improving HFrEF is frequently obscure in real-world settings because the standard cardioprotective approach (diuretics, β-blockers, and angiotensin-converting enzyme inhibitors/angiotensin II receptor blockers) is essential for the initial treatment of HFrEF. Eight of nine patients showed improvement in cardiac function with osimertinib discontinuation, albeit with the cardioprotective approach, but one died in four weeks despite the above measures [8]. In that case, a coronary angiogram showed negative results, and the author concluded that lethal cardiomyopathy was associated with osimertinib. Follow-up TTE showed partial or full restoration of LVEF in seven cases except for the lethal case. It should be noted that the time to LVEF recovery after drug discontinuation varied widely among cases. While LVEF was not evaluated serially in these studies, previous studies reported that LVEF recovered in weeks to months (Table 1). Our patient had the earliest LVEF recovery after one week.

Rechallenging the drug would be another approach to assume the drug-induced cardiomyopathy, despite an unclear safety profile for osimertinib. Only two subjects resumed osimertinib following the HFrEF, and the cardiac failure did not recur [8,9]. Conversely, recurrence of osimertinib-associated cardiomyopathy due to re-challenge was repeated in a patient with asymptomatic LVEF reduction [8]. In the present case, the patient refused to resume osimertinib treatment and opted for standard supportive care.

## Conclusions

In summary, we presented a case of symptomatic HFrEF following osimertinib administration in a patient with advanced lung cancer. Large-scale clinical studies have suggested that osimertinib-induced cardiomyopathies are infrequent and mostly subclinical. On the contrary, real-world patients rarely develop symptomatic HFrEF in the relatively early phase. Physicians should be aware of rare complications associated with osimertinib therapy.
